# 

**Published:** 1899-06

**Authors:** 


### SELECTIONS.

Article I.—Concerning the Possibilities in the Art of Filling Teeth. H. A. Smith, D. D. S .................. 49

" II.—Inflammation of the Perodontal Membrane, Periodontitis, Alveola Abscess, Necrosis, and Other Sequelæ. R. Denison Pedley, M. R. C. S , L. D. S ............ 63

" III.—Pyorrhœa Alveolaris. M. L. Rhein, M. D., D. D. S... 69

" IV.—Extract from an Article upon "Some Overlooked Sources of Infection. M. L. Dyer ................ 83

" V.—Do Medical Colleges Strive to Thoroughly Educate Their Students? G. Lennox Curtis, M. D ......... 84

" VI.—Concerning Fees ........................... 86

### COMMUNICATED.

National Association of Dental Examiners..................... 88

National Dental Association ........................................ 88

### BIBLIOGRAPHICAL.

A Dictionary of Dental Science and Such Words and Phrases of the Collateral Sciences as Pertain to Art and Practice of Dentistry. 90

### MONTHLY SUMMARY.

Dr. Black's Theory........................................ 91

Dentifrice Soap........................................ 91

Supraorbital Neuralgia ........................................ 92

The Insertion of Dentures ........................................ 92

To Clean an Oil Stone........................................ 92

Does License to Practice Medicine Permit the Practice of Oral Surgery........................................ 93

Soldering Gold Crowns........................................ 93

Cinnamon Water as a Dressing........................................ 94

Epilepsy ........................................ 95

Substitute for Rubber Gloves........................................ 96

Bridge or Plate? ........................................ 96

